# From the “top-down” and the “bottom-up”: Centering Foucault’s notion of biopower and individual accountability within systemic racism

**DOI:** 10.1007/s40037-021-00655-y

**Published:** 2021-02-24

**Authors:** Zareen Zaidi, Antonio A. Bush, Ian M. Partman, Tasha R. Wyatt

**Affiliations:** 1 Department of Medicine, University of Florida College of Medicine, 103204, 32610 Gainesville, FL USA; 2 Equity, Diversity, and Inclusion, Association of American Medical Colleges, Division of Practice Advancement and Clinical Education, UNC Eshelman School of Pharmacy, University of North Carolina at Chapel Hill, Chapel Hill, NC USA; 3 New York University, NY, NY USA; 4 Educational Innovation Institute, Medical College of Georgia, Augusta University, Augusta, GA USA

In the wake of worldwide events coalescing in 2020, the presence of anti-Black racism in the United States was made visible to those abroad and its egregiousness made more explicit to some citizens previously unaware of it in the U.S. In addition to the onset of the COVID-19 pandemic exposing deep-seated structural health disparities between white and non-white communities, a global mass uprising emerged in response to George Floyd’s death [1, 2]. In ways that could not have been anticipated even a few years earlier, segments of American society have had to reckon with the pervasive, powerful forces of white supremacy and the ways society and its structures have disadvantaged racially minoritized groups. In this wide-sweeping shift, medical education and medicine have also grappled with these issues, especially the ways in which medical education perpetuates institutional racism.

Presently, American society disadvantages non-white individuals in ways that have guaranteed their “skewed life chances, limited access to health and education, premature death, incarceration, and impoverishment” [3, p. 3]. These deep-seated societal disadvantages have existed for centuries as a result of Western engagement in slavery and its aftermath. This problem endured throughout the last century (and beyond), exemplified in 1903 by W.E.B. Du Bois’s oft-quoted statement, “The problem of the twentieth century is the problem of the color line” [4, p. xii]. This quote, from Du Bois’s “The Souls of Black Folk,” is a “rallying cry” for the oppressed, and “demonstrates unequivocally how the race problem is forever linked to the American Dream” [4, p. xiii]. Thus, it portrays how racism should be a matter for those all over the world, but contextualized specifically for the Black citizens in the United States who have felt the wrath of racism, as well as those who knowingly and unconsciously upholds its structures. As such, Du Bois exclaims, “The burden belongs to the nation, and the hands of none of us are clean if we bend not our energies to righting these great wrongs” [5]. At present, the “color line,” referencing the divide between races, continues to be frontstage in the U.S. and globally as demonstrated, most recently, with the white supremacist insurrection occurring at the nation’s Capitol building in the U.S. [6]. Appropriately, within the past year, societies across the globe have been engaged in conversations around race and racism, and the ways in which these forces shape society, and the experiences of racially minoritized individuals living within them.

To widen this issue as to how racism is perpetuated in medical education, we look to the philosopher Michel Foucault, who asserts that power is everywhere [7]. In his work, he coined the term *biopower*—literally to have power over others’ bodies—to describe how institutions exert power over the human body in the name of the social good [8, p. 140]. In his work, Foucault examined the numerous ways the human body is manipulated and used in the context of a particular social system. For example, Foucault analyzed how discourse reinforces norms around sexuality, sanity versus insanity, and how the framing of these issues allows individuals to be manipulated and controlled by outside forces. In creating categories of what is “normal” for/in the human body, bodies become docile and are used to exclude individuals who do not fit socially acceptable understandings or categories.

From a Foucauldian perspective, power is not just an interaction occurring at a macro-level whereby governments or institutions exert influence on society. Rather, power is also enforced on the micro-level by individuals between doctors and patient, teachers and students, etc. It is maintained through these dispersed, decentralized networks, which Foucault calls the *dispositif *[9]. In this conceptualization, power is dissipated throughout institutions by individual actors (i.e., teachers, administrators, and students) who contribute to the creation and maintenance of institutional norms and practices. For example, when medical educators only show photos of white skin when discussing dermatological issues, according to Foucault these preferential acts normalize the presentation of the disease in white skin, which in turn disadvantages patients with a different skin color. In this way, preferences for what is normal are perpetuated throughout the profession in ways that frequently go unaccounted for.

In contrast to other conceptualizations of power, which begin at the macro-levels of society and trickle down into the micro-levels, biopower begins at the micro-level, exerting itself in everyday life. Foucault’s conceptions are important to consider because systemic racism operates at the macro-level, in which public policies, institutional practices, cultural representations, and other norms work in various and reinforcing ways to perpetuate racial inequity [10]. Focusing on systemic racism is important because it identifies dimensions of our history and culture that have allowed privileges associated with “whiteness” to endure and adapt over time, while continuing to disadvantage groups associated with “color.” At the same time, these structures are comprised of an amalgamation of individuals who work to keep the institution in operation. They are products of a society that is imbued with whiteness and therefore bring their lenses, tools, and decision-makings capability to the medical education enterprise. In this way, not only are institutions contributing to the problem, but so are the people within them. Thus, racism is being boundlessly perpetuated from both the “top-down” in public policies, institutional practices, cultural representations, and other norms and the “bottom-up” in the individuals working within these systems. For this reason, Foucault’s work urges us to not only focus on systemic racism at the macro-level, but to also address it at the micro-level, by considering the ways biopower—power over others’ bodies, including the ways bodies are represented, talked about, counted, rendered highly visible/invisible—is perpetuated in formal and informal ways to maintain white supremacy.

In a recent *Perspectives’* article included in this issue, a group of students representing several centers in the U.K. described some of the mechanics of institutional racism embedded in healthcare and medical education [11]. They argue that medical education’s portrayal of race is a vital component needed to better prepare physicians to care for marginalized communities. The authors draw attention to the ways race is positioned and obscured in health, such as physicians’ miscalculations in assessing pain in Black patients, the erroneous ways physiological tests are adjusted for Black patients (e.g., kidney function), educators’ silence on racial issues in curricular content, and medical education’s testing practices that reinforce racial stereotypes. In addressing these issues, Lim et al. propose changes to help facilitate a deconstruction of institutional racism in medical education and healthcare [11].

Their suggestions include updating medicine’s educational material and question banks, which typically reinforce a biological understanding of race and overlooks it as a social construct. Making this shift to understanding race as a social construction is important because it affords physicians the opportunity to address racialized health disparities and look to larger contextual issues that contribute to poor health outcomes [12]. And yet, while these suggestions are helpful in drawing attention to larger issues showing how medical education frames and reinforces problematic understandings of race, by only focusing on curricula’s contribution, individuals working within these systems are effectively unaccountable for their role in perpetuating this system.

## Moving forward to include biopower

Throughout medical education there have been calls for going beyond cultural competence and cultural humility to focus on institutions, systems, and policies to address health disparities and racism [13–16]. While a wider view of how society enables harmful practices that disadvantage non-whites is crucial, it is also important to continue with measures to create awareness in the *dispositif* within institutions. One such call has been to question and reimagine pre-existing curricular and pedagogical models that are taken for granted in perpetuating systemic racism [11]. For example, didactic instruction, small group learning, medical simulation, and accreditation processes leave little space to consider the effects of racism and historical trauma and their role in health inequities and advocacy. Rather, biomedical content is foregrounded leaving little consideration of the lived experiences and sociohistorical context surrounding patients, rendering invisible how social location, medical context, and lived experiences influence one’s health [15].

Further, while it is imperative that we meet each individual where they are on their journeys to becoming anti-racist, programs such as cultural competency, racial sensitivity, and diversity training alone are inadequate to alleviate institutional failings. For medical education to be anti-racist, the current state of medical education must be examined and new pedagogical practices created to ensure that the sociohistorical contexts of non-whites are not excluded from students’ training experiences [17]. Embedded in this anti-racism process must be a collective effort to go beyond a lack of awareness to fostering a desire of motivation “*to want to, rather than have to*, engage in the process of becoming culturally aware, culturally knowledgeable, culturally skillful, and familiar with cultural encounters” [16, p. 182]. It begins with teaching educators to implement critical pedagogy, which is a method of teaching that describes how and why some constructions of reality are legitimated while others are not, as well as how everyday commonsense understandings (i.e., social constructions) are produced [18]. It means that the very act of teaching must be re-packaged as a combination of education, pedagogy, and assessment that are cultural and political acts used in both regimes of racial oppression and dominance.

As a profession, we must also go beyond simply cultivating an understanding of racism in medicine to develop solutions that rectify and dismantle the institutions which make such a lack of understanding possible [17]. Ultimately, it means that we need to empower students, staff, faculty, and administrators to be able to resist practices and regulations in medical education that render racism as invisible, rather than merely conforming to the system that has been handed down to them. Focusing on the individual’s responsibility in shaping and changing medical education helps to refocus power as much as an individual arrangement, as an institutional one. Because, as Du Bois would suggest, as institutional members we are all implicated in racism and white supremacy, and the sooner we take responsibility for this, the sooner we can get to work on creating change. As such, we leave you with a set of Foucauldian-like questions: How are you upholding the “color line”? What is your role in eradicating the divisiveness perpetuated through the “color line”?

## References

[1] Yancy CW (2020). COVID-19 and African Americans. JAMA.

[2] Ehrenfeld JM, Harris PA. Police brutality must stop. 2020. https://www.ama-assn.org/about/leadership/police-brutality-must-stop. Accessed 23 Nov 2020.

[3] Hartman S (2008). Lose your mother: a journey along the Atlantic slave route.

[4] Du Bois WEB (1990). The souls of black folk. Library of America edition.

[5] Thomas J. The problem of the twentieth century is the problem of the color-line. 2021. https://oaklandpostonline.com/34148/opinion/the-problem-of-the-twentieth-century-is-the-problem-of-the-color-line/. Accessed 23 Nov 2020.

[6] Borger J. Insurrection Day: when white supremacist terror came to the US Capitol. 2021. https://www.theguardian.com/us-news/2021/jan/09/us-capitol-insurrection-white-supremacist-terror. Accessed 16 Jan 2021.

[7] Foucault M (1977). Discipline and punish: the birth of the prison.

[8] Foucault M (1978). The history of sexuality.

[9] Foucault M (2007). Security, territory, population: lectures at the Collège de France, 1977–78.

[10] Hardeman RR, Medina EM, Kozhimannil KB (2016). Structural racism and supporting black lives—the role of health professionals. N Engl J Med.

[11] Lim GHT, Sibanda Z, Erhabor J, Bandyopadhyay S (2021). Students’ perceptions on race in medical education and healthcare. Perspect Med Educ.

[12] Sharma M, Kuper A (2017). The elephant in the room: talking race in medical education. Adv Health Sci Educ Theory Pract.

[13] Neff J, Holmes SM, Knight KR, Strong S, Thompson-Lastad A, McGuinness C (2020). Structural competency: curriculum for medical students, residents, and interprofessional teams on the structural factors that produce health disparities. MedEdPORTAL.

[14] Gray DM, Joseph JJ, Glover AR, Olayiwola JN (2020). How academia should respond to racism. Nat Rev Gastroenterol.

[15] Dao DK, Goss AL, Hoekzema AS, Kelly LA, Logan AA, Mehta SD (2017). Integrating theory, content, and method to foster critical consciousness in medical students: a comprehensive model for cultural competence training. Acad Med.

[16] Campinha-Bacote J (2002). The process of cultural competence in the delivery of healthcare services: a model of care. J Transcult Nurs.

[17] Paton M, Naidu T, Wyatt TR, Oni O, Lorello GR, Najeeb U (2020). Dismantling the master’s house: new ways of knowing for equity and social justice in health professions education. Adv Health Sci Educ Theory Pract.

[18] Halman M, Baker L, Ng S (2017). Using critical consciousness to inform health professions education. Perspect Med Educ.

